# Lymphocyte proliferation induced by high-affinity peptides for HLA-B*51:01 in Behçet’s uveitis

**DOI:** 10.1371/journal.pone.0222384

**Published:** 2019-09-12

**Authors:** Toshikatsu Kaburaki, Hisae Nakahara, Rie Tanaka, Kimiko Okinaga, Hidetoshi Kawashima, Youichiro Hamasaki, Thanyada Rungrotmongkol, Supot Hannongbua, Hiroshi Noguchi, Makoto Aihara, Fujio Takeuchi

**Affiliations:** 1 Department of Ophthalmology, The University of Tokyo Graduate School of Medicine, Tokyo, Japan; 2 Department of Ophthalmology, Jichi Medical University, Tochigi, Japan; 3 Department of Dermatology, Dokkyo Medical University, Tochigi, Japan; 4 Department of Biochemistry, Faculty of Science, Chulalongkorn University, Bangkok, Thailand; 5 Computational Chemistry Unit Cell, Department of Chemistry, Faculty of Science, Chulalongkorn University, Bangkok, Thailand; 6 Department of Pharmacognosy, Nihon Pharmaceutical University, Saitama, Japan; 7 Department of Pharmacology, University of Shizuoka, Shizuoka, Japan; 8 Department of Health and Nutrition, Tokyo Seiei University, Tokyo, Japan; Université Paris Descartes, FRANCE

## Abstract

Several proteins have been proposed as candidate auto-antigens in the pathogenesis of Behçet’s disease (BD). In this study, we aimed to confirm the cellular responses to candidate peptide autoantigens with high affinity for the HLA-B*51:01 molecule using computerized binding predictions and molecular dynamics simulations. We identified two new candidate peptides (HSP65PD, derived from heat shock protein-65, and B51PD, derived from HLA-B*51:01) with high-affinity to the HLA-B*51:01 binding pocket using the Immune Epitope Database for Major Histocompatibility Complex-I Binding Prediction and molecular dynamics simulations. The peptide-induced proliferation of lymphocytes from patients with BD, sarcoidosis, Vogt–Koyanagi–Harada disease (VKH) with panuveitis, systemic scleroderma (SSc) without uveitis, and healthy controls (HC) was investigated using the bromodeoxyuridine assay. The proliferative response of leukocytes to HSP65PD was significantly higher in BD (SI 1.92 ± 0.65) than that in sarcoidosis (SI 1.38 ± 0.46), VKH (SI 1.40 ± 0.33), SSc (SI 1.32 ± 0.31), and HC (SI 1.27 ± 0.28) (P = 0.0004, P = 0.0007, P < 0.0001, P < 0.0001, respectively, Mann-Whitney’s U-test). The proliferative response of leukocytes to B51PD was also higher in BD than that in sarcoidosis, VKH, SSc without uveitis, and HC, whereas no significant differences were observed among the five groups in response to a control peptide derived from topoisomerase 1. A significantly higher response to HPS65PD and B51PD was observed in the HLA-B*51:01-positive patients with BD than in the HLA-B*51:01-negative patients. In conclusion, two peptides that had high affinity to HLA-B*51:01 in computerized binding prediction showed significantly higher response in HLA-B*51:01-positive patients with BD, indicating the usefulness of computerized simulations for identifying autoreactive peptides to HLAs.

## Introduction

Behçet’s disease (BD), a multi-organ inflammatory disease characterized by recurrent uveitis, oral aphthous stomatitis, genital ulcers, and skin lesions, is more prevalent in countries along the ancient “Silk Road” from Japan to the Mediterranean region than elsewhere in the world. Although the pathogenesis of BD is not well understood, genetic factors, especially human leukocyte antigens (HLA), are believed to play an important role in the pathogenesis of this disease [1, 2]. T-cell mediated immune reactions may also be associated with this disease as patients with active BD had significantly more IFNγ-producing CD4+ cells [3] and activated CD8+ and γδ T cells [4] than those in inactive cases and control subjects. Moreover, the frequencies of Th1/Th17 cells in peripheral blood were increased in BD with active uveitis [5] and the alterations of T cell balance, especially increase of T helper 1 (Th1) and Th17 cells and decrease of regulatory T (Treg) cells, are considered to have a significant role in the pathogenesis of BD [6].

T cells recognize a complex composed of a major histocompatibility complex (MHC) molecule and an antigenic peptide or epitope. The identification of pathogenic antigens or antigenic epitopes is crucial for facilitating the study of immune-mediated inflammatory diseases. Several studies have demonstrated that peripheral blood lymphocytes (PBL) from patients with BD and panuveitis are sensitized to retinal S-antigen (S-Ag), and autoreactive lymphocytes to S-Ag or its peptide motif were present in the peripheral blood of patients with BD [7,8]. Furthermore, several candidate antigens or their peptide motifs [9], such as major histocompatibility complex class I chain-related gene A (MICA) [4], heat shock protein-65 (HSP65) [10], retinal S-antigen [11], and alpha-tropomyosin [12], have been reported to elicit response from PBL of patients with BD. In addition, peptides derived from the HLA-B*51:01 molecule itself have a predicted binding capacity to HLA-B*51:01 [9, 13]. Although the high prevalence of HLA-B51 in BD suggested the importance of this HLA type in the pathogenesis of BD, the putative role of these candidate antigens and HLA-B51 in the pathogenesis of BD is still obscure.

The accuracy of predicting peptide-MHC binding is useful for candidate T cell epitope selection as it minimizes the number of experiments required for their identification. The binding groove of class I MHC can accommodate 8–11 amino acid-long short peptides [14]. Sequence motifs are still the best-known tools for predicting the peptide specificity of allele-dependent MHC peptide binding. Currently, the MHC-I binding for any peptide sequences is predicted using the Immune Epitope Database (IEDB) analysis resource Consensus tool [15], which combines predictions from ANN aka NetMHC (4.0) [16–18], SMM [19], and Comblib [20], all of which are freely available on the IEDB website (http://www.immuneepitope.org).

Recently, using molecular dynamics simulations, we demonstrated that a 9-mer peptide, MICA-TM, which had been reported to induce proliferation of PBL from patients with BD, had a significantly stronger total binding free energy with the BD-associated HLA alleles (HLA-B*51:01 and A*26:01) than with the non-associated alleles (HLA-B*35:01 and A*11:01) [21]. This result suggests that structure, dynamics, and energetics may explain the recognition and selective binding of immunogenic peptides to specific HLAs. Thus, computerized binding predictions of peptide motifs with high affinity to HLA [15] or computational dynamics simulation of the bonding energy between HLA and candidate peptides [21] may select and provide T cell epitopes to induce T cell proliferation in BD effectively.

In the present study, we investigated the proliferation of PBL from patients with BD patients and panuveitis along with that in non-BD patients induced by newly determined candidate peptides that have high affinity to HLA-B*51:01 using the IEDB analysis resource Consensus tool [15].

## Materials and methods

### Patients

A total of 47 patients with BD and panuveitis who met the diagnostic criteria established by the Behçet’s Disease Research Committee of Japan [22] and were followed at the outpatient uveitis clinic of the University of Tokyo Hospital were studied. Patients with other forms of non-infectious panuveitis (*n* = 37; 19 cases of sarcoidosis and 17 cases of Vogt–Koyanagi–Harada disease (VKH)) and 15 patients with systemic inflammatory disease without uveitis and systemic scleroderma (SSc) acted as controls. Furthermore, 17 age- and sex-matched healthy individuals (healthy control, HC) were enrolled in this study as negative controls. The diagnosis of sarcoidosis [23], VKH [24], and SSc [25] was based on the respective specific diagnostic criteria. The inclusion criteria for BD and other non-infectious panuveitis in this study were as follows; (1) patients with panuveitis who had recurrence of uveitis within 6 months, or (2) patients with active systemic inflammatory signs owing to the disease (i.e., oral aphthae in BD and granulomatous skin lesions in sarcoidosis). The patients were enrolled in this study irrespective of their current medical therapy and disease severity. This study was approved by the ethical committees of the University of Tokyo Hospital (G10136) and written informed consent for participating in this study was obtained from all participants prior to blood collection.

The severity of ocular inflammation of uveitis in the inflamed eyes and use of systemic corticosteroid and immunosuppressants at blood collection were retrospectively examined from the clinical records. The severity of ocular inflammation was graded according to the criteria established by the Standardization of Uveitis Nomenclature (SUN) Working Group [26]. Briefly, cells in the anterior chamber (maximum 4 points) were graded using a semi-quantitative scoring system of 6 grades (0, 0.5+, 1+, 2+, 3+, and 4+). Vitreous haze (maximum 4 points) was evaluated using a semi-quantitative scoring system of 6 grades (0, trace, 1+, 2+, 3+, and 4+) based on the clarity of the optic disc, retinal vessels, and nerve fiber layers in fundus examination reported by Nussenblatt et al. [27]. For convenience, vitreous haze graded as trace in the Nussenblatt grading [27] was noted as 0.5+ in this study. The severity of uveitis was scored in the severe inflamed eye if both eyes were inflamed at blood collection.

### Selection and synthesis of antigenic peptides

Several candidate peptide sequences with potentially high binding capacity to HLA-B*51:01 determined using bioinformatics programs [15] have been previously reported [9]. In the present study, we focused on two peptides, HSP65PD and B51PD, derived from HSP65 and HLA-B*51:01, respectively, as they were speculated to have high binding capacity to HLA-B*5101 [9] and the proliferation of lymphocytes from patients with BD against these peptide sequences had not been previously investigated. A well-known peptide for MICA (MICA-TM), which induced significant proliferation of T lymphocytes from patients with BD carrying HLA-B*51:01 was used as the positive control [4, 21]. A peptide derived from topoisomerase 1 (Topo1PD), a specific autoreactive antigen in patients with SSc, was used as the negative control [28, 29]. Recently, molecular dynamics simulation revealed that a 20-mer peptide sequence of topoisomerase 1 showed significantly high binding to HLA-DR subtypes associated with anti-topoisomerase 1 (or anti scl-70) antibody-positive SSc (HLA-DRB1*08:02, HLA-DRB1*11:01, and HLA-DRB1*11:04) [30]. The peptide sequences in the present study and their affinity to HLA-B*51:01 calculated using the IEDB analysis resource Consensus tool [15] are shown in Table 1.

**Table 1:** Candidate peptides and binding affinity to HLA*B5101.

Peptide name	Protein	Length	Sequence	Percentile Rank in IEDB analysis	ann_ic50	ann_ rank	smm_ic50	smm_ rank	comblib_sidney 2008_score	comblib_sidney 2008_rank
HSP65PD	Heat shock protein 65	9	SALQNAASI	1.6	7822.84	1.6	2751.88	1.6	6.15E-05	4.1
B51PD	HLA*B5101	9	RAYLEGLCV	4.6	8197.79	1.7	6525.74	4.6	0.00054	32
MICA-TM	MICA	9	AAAAAIFVI	1.8	8396.09	1.8	6034.34	4.2	1.09E-05	0.6
Topo1PD	Topoisomerase 1	9	FKIEPPGLF	10	17558.43	6	13951.79	10	0.00129	54

The half maximal inhibitory concentration (IC50) is a measure of the potency of the peptide motif in inhibiting the specific binding of the radio-labeled peptide to HLA-B*5101.

MICA, major histocompatibility complex class I chain–related gene A; IEDB, the Immune Epitope Database and Analysis

Resource; IC50, half maximal (50%) inhibitory concentration; ann, artificial neural networks; smm, stabilized matrix method

comblib_sidney2008, combinatorial library approach by Sidney et al. [20].

### Separation of PBL and proliferation assay

PBL from patients and controls were isolated via centrifugation on a Ficoll-Hypaque gradient for 20 min, washed twice, and resuspended in Roswell Park Memorial Institute 1640 medium with HEPES, supplemented with 2 mM glutamine (Sigma-Aldrich Japan, Tokyo, Japan), 100 units/ml penicillin G (Sigma-Aldrich Japan, Tokyo, Japan), 100 μg/ml streptomycin sulfate, and 10% heat-inactivated human AB serum (Biowest, Nuaillé, France). Cells (1 × 10^5^/well) were incubated in a total volume of 100 ml with the peptides or concanavalin A (4 μg/ml, Fujifilm Wako Pure Chemical Co., Osaka, Japan) in triplicates in 96-well round-bottom culture plates (Nunc, Roskilde, Denmark) at 37°C in a humidified atmosphere with 5% CO_2_. The cultures were incubated for 4 days and then pulsed with bromodeoxyuridine (BrdU) during the last 16 h of incubation. Cellular proliferation was assessed using a BrdU enzyme-linked immunosorbent assay kit (Cell Proliferation ELISA BrdU, Sigma), according to the manufacturer’s instructions. After co-culture with Brdu for 16 h, the BrdU labeling solution was removed and anti-BrdU antibody was added. The OD at 450 nm for BrdU was measured using a luminescence plate reader (ARVO X3, Perkin Elmer Japan, Yokohama, Japan). The results were expressed as stimulation index (SI: mean OD_450nm_ in stimulated cultures / mean OD_450 nm_ in unstimulated control cultures).

### Statistical analysis

Lymphocyte proliferation was analyzed using the Mann–Whitney U-test. P value < 0.05 was considered statistically significant. Mean and standard deviation (SD) values are shown in the text. Statistical analysis was performed using SPSS for Windows version 23.0 (IBM Corp., Armonk, NY, USA).

## Results

### Patient characteristics

The demographics of the participants of this study are shown in Table 2. The ages of the patients ranged between 21 and 81 years, and the mean age for each disease varied as the peak age of onset for each disease was different. Sex distribution was also considerably different for the same reason. Male sex was more frequent in patients with BD than in those with control uveitis (sarcoidosis and VKH) and SSc (P < 0.0001, Chi-square test). However, there were no significant differences in the age and sex distribution between BD and HC (P = 0.226, Chi-square test). The duration of the disease was longer in patients with BD, probably because patients with relatively severe disease showed recurrence of uveoretinitis despite administration of systemic immunosuppressants.

**Table 2:** Patient demographics.

	BD	Sar	VKH	SSc	HC
Number of cases	47	19	17	15	17
Mean age (range)	46.2 (22–66)	60.1 (21–77)	53.6 (26–78)	63.6 (21–81)	42.3 (27–65)
Male/Female	35/12	3/16	12/6	0/15	10/7
Duration of disease (years)	11.3 (0–24)	8.0 (0–21)	6.3 (0–15)	6.7 (0–26)	-
HLA-B*51:01 positive/negative	20/27 (42.6%)	-	-	-	-
HLA-A*26 positive/negative	24/23 (51.1%)	-	-	-	-
Anti-topoisomerase 1 antibody positive/negative	-	-	-	4/15 (26.7%)	-
Ocular inflammation scores in inflamed eye at blood collection					
Cells in anterior chamber	0.28 ± 0.62	0.37 ± 0.57	0.47 ± 0.86	-	-
Vitreous haze	0.28 ± 0.62	0.39 ± 0.58	0.21 ± 0.40	-	-
Medications at blood sampling					
Corticosteroid (mg/day)	0.89 ± 2.19	2.53 ± 4.25	4.38 ± 4.25	2.93 ± 5.83	-
Cyclosporine (mg/day)	35.3 ± 68.1	0 ± 0	57.4 ± 92.2	0 ± 0	-
TNF inhibitors (cases, %)	23 (48.9%)	2 (10.5%)	5 (29.4%)	0 (0%)	-

BD, Behçet's disease; Sar, sarcoidosis; VKH, Vogt–Koyanagi–Harada disease; SSc, scleroderma; HC, healthy control; HLA, human leukocyte antigen; TNF, tumor necrosis factor

The grades of ocular inflammation in anterior chamber cells in the inflamed eyes at blood collection were 0.28 ± 0.62 in BD, 0.37 ± 0.57 in sarcoidosis, and 0.47 ± 0.86 in VKH. Furthermore, the severity scores of vitreous haze were 0.28 ± 0.62 in BD, 0.39 ± 0.58 in sarcoidosis, and 0.21 ± 0.40 in VKH (Table 2). There were no significant differences in the scores in anterior chamber cells and vitreous haze at blood collection among the three uveitis groups (P = 0.345 and P = 0.245, Kruskal-Wallis test). Systemic corticosteroid was administrated at blood sampling in 8 cases of BD (17.0%, 0.89 ± 2.19 mg), 7 cases of sarcoidosis (36.8%, 2.53 ± 4.25 mg), 10 cases of VKH (58.8%, 4.38 ± 4.25 mg), and 4 cases of SSc (26.7%, 2.93 ± 5.83 mg). Moreover, usage of TNF inhibitor (infliximab or adalimumab) was more frequent in BD (48.9%) than in sarcoidosis (10.5%), VKH (29.4%), and SSc (0%).

### Responses to candidate peptides

**Fig 1:**
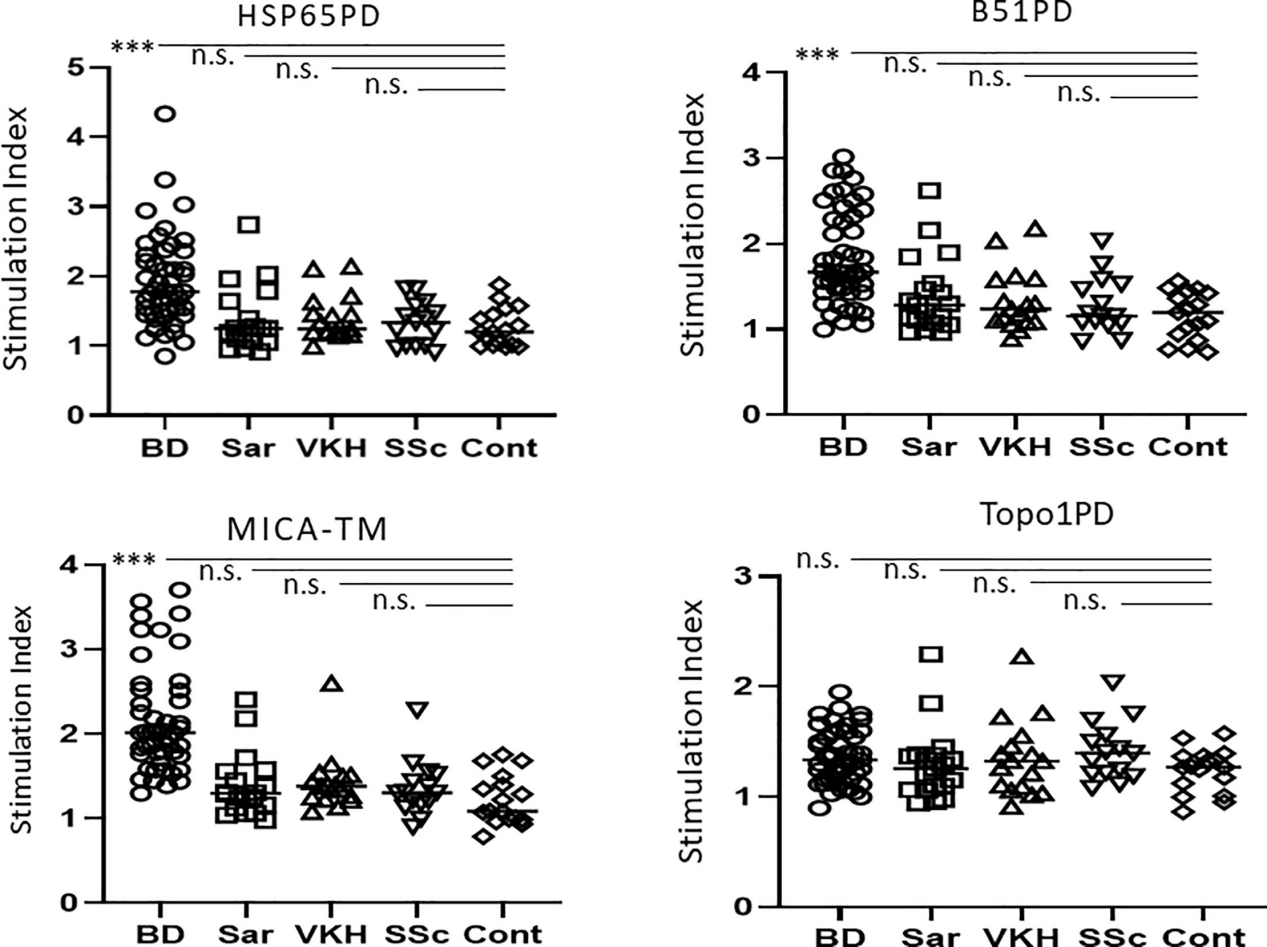
Candidate peptide-induced lymphocyte proliferation in patients with uveitis. Lymphocyte proliferative responses to four peptides (HSP65, B51PD, MICA-TM, and Topo1PD) were investigated using bromodeoxyuridine assays. The SI for each group is shown. The statistical differences between HC and Behçet's disease, sarcoidosis, Vogt-Koyanagi-Harada disease, and scleroderma were determined using Mann–Whitney’s U-test. n.s., Not significant; **P < 0.001. BD, Behçet's disease; Sar, sarcoidosis; VKH, Vogt–Koyanagi–Harada disease; SSc, scleroderma; HC, healthy control.

The proliferation reactions of PBL from patients with BD after stimulation by candidate peptides (HSP65PD and B51PD) and control peptides (MICA-TM and Topo1PD) were compared to those of PBL from disease controls (sarcoidosis, VKH, SSc) and HC (Fig 1). Patients with BD had significantly higher lymphocyte proliferation responses to HSP65PD (SI 1.92 ± 0.65) than did HC (SI 1.27 ± 0.28) (P = 0.0004, Mann–Whitney’s U-test), whereas the lymphocyte responses of those with sarcoidosis (SI 1.38 ± 0.46), VKH (SI 1.40 ± 0.33), and SSc (SI 1.32 ± 0.31) to HSP65PD were not significantly higher than the response of HC (SI 1.27 ± 0.28). The responses to B51PD were also significantly stronger in patients with BD (SI 1.85 ± 0.55) than those in HC (SI 1.27 ± 0.28) (P = 0.001, Mann–Whitney’s U-test), whereas responses in patients with sarcoidosis (SI 1.40 ± 0.45), VKH (SI 1.40 ± 0.33), and SSc (SI 1.32 ± 0.31) were similar to those in HC (SI 1.27 ± 0.28) (n.s. in HC vs. sarcoidosis, VKH, and SSc, respectively, Mann–Whitney’s U-test).

As a positive control, the proliferative response to MICA-TM was assessed. The SIs for BD, sarcoidosis, VKH, SSc, and HC were 1.85 ± 0.55, 1.40 ± 0.45, 1.40 ± 0.33, 1.32 ± 0.31, and 1.27 ± 0.28, respectively (P < 0.0001, n.s., n.s., n.s. for HC vs. BD, sarcoidosis, VKH, and SSc, respectively, Mann–Whitney’s U-test). The corresponding SIs for the negative control peptide, Topo1PD, were 1.37 ± 0.31, 1.27 ± 0.34, 1.35 ± 0.34, 1.41 ± 0.26, and 1.24 ± 0.20, respectively. The peptide Topo1PD did not significantly increase the response of PBL from patients with BD, sarcoidosis, VKH, and SSc, compared to those from HC (n.s., in HC vs. BD, sarcoidosis, VKH, and SSc, respectively, Mann–Whitney’s U-test). We also examined the effect of sex on proliferation of PBL in response to the peptides in patients with BD. However, there were no significant differences according to sex on lymphocyte proliferation (S1 Fig).

### Association of HLA-B*51:01 and T cell proliferation

**Fig 2:**
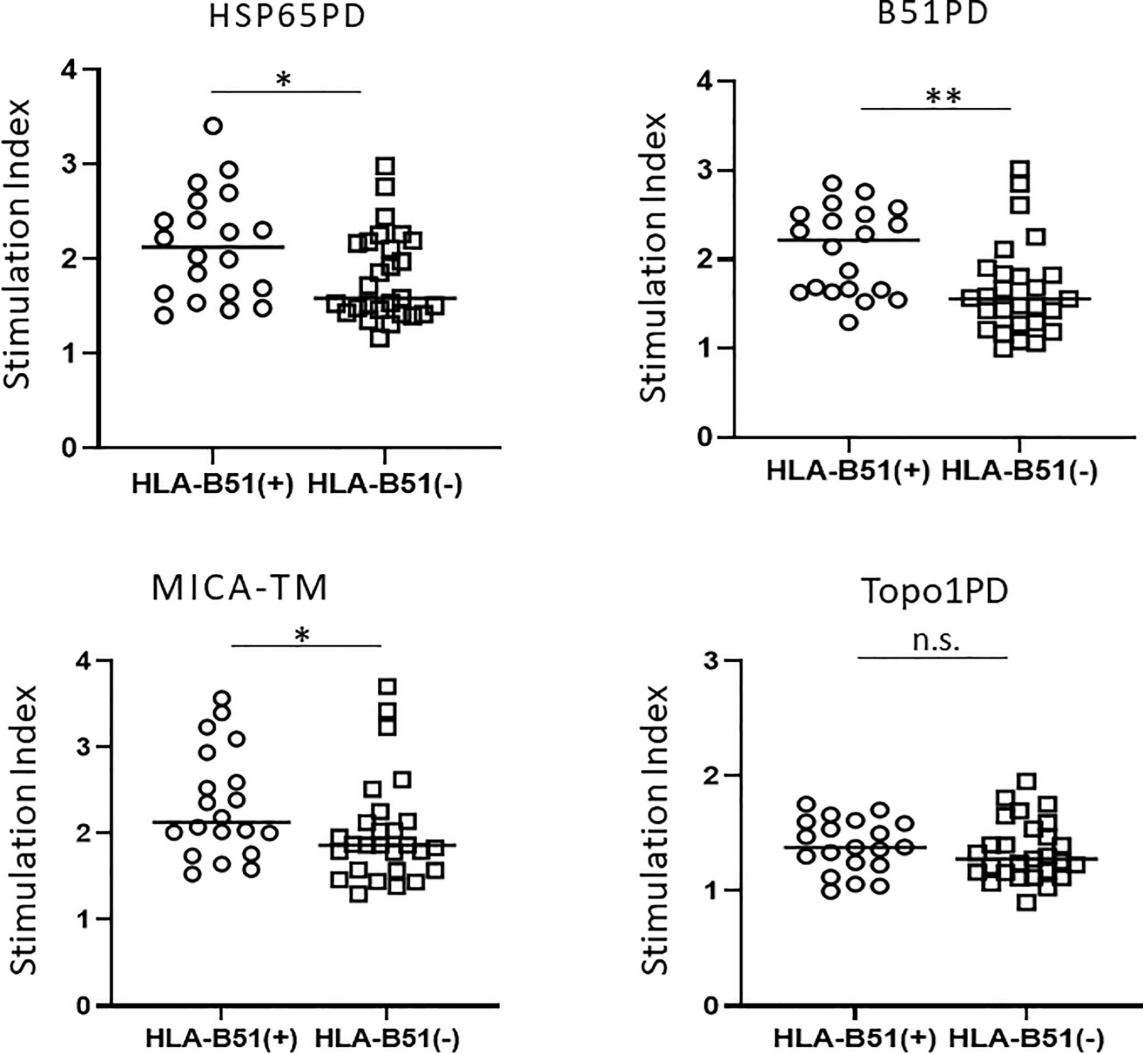
Association of HLA-B*51:01 and T cell proliferation in Behçet’s disease. Lymphocyte proliferative responses in Behçet's disease induced by four peptides (HSP65, B51PD, MICA-TM, and Topo1PD) were compared between human leukocyte antigen (HLA)-B*51-positive patients and HLA-B*51:01-negative patients. The SI for each group is shown. The statistical significances between BD and other diseases were determined using Mann–Whitney’s U-test. n.s., Not significant; *P < 0.05. HLA, human leukocyte antigen.

Next, we investigated the association of HLA-B*51:01 in patients with BD and T cell proliferation induced by candidate peptides (Fig 2). The SI was significantly higher in the HLA-B*51:01-positive BD group than that in the HLA-B*51:01-negative BD group when the PBL were stimulated by HSP65, B51PD, and MICA-TM (P < 0.05, P < 0.05, P < 0.05, respectively, Mann–Whitney’s U-test). However, there was no significant difference in PBL proliferation between HLA-B*51:01-positive and -negative BD groups when the PBLs were stimulated by Topo1PD, the control peptide (n.s., Mann–Whitney’s U-test).

### Association of HLA-A*26 and T cell proliferation

**Fig 3:**
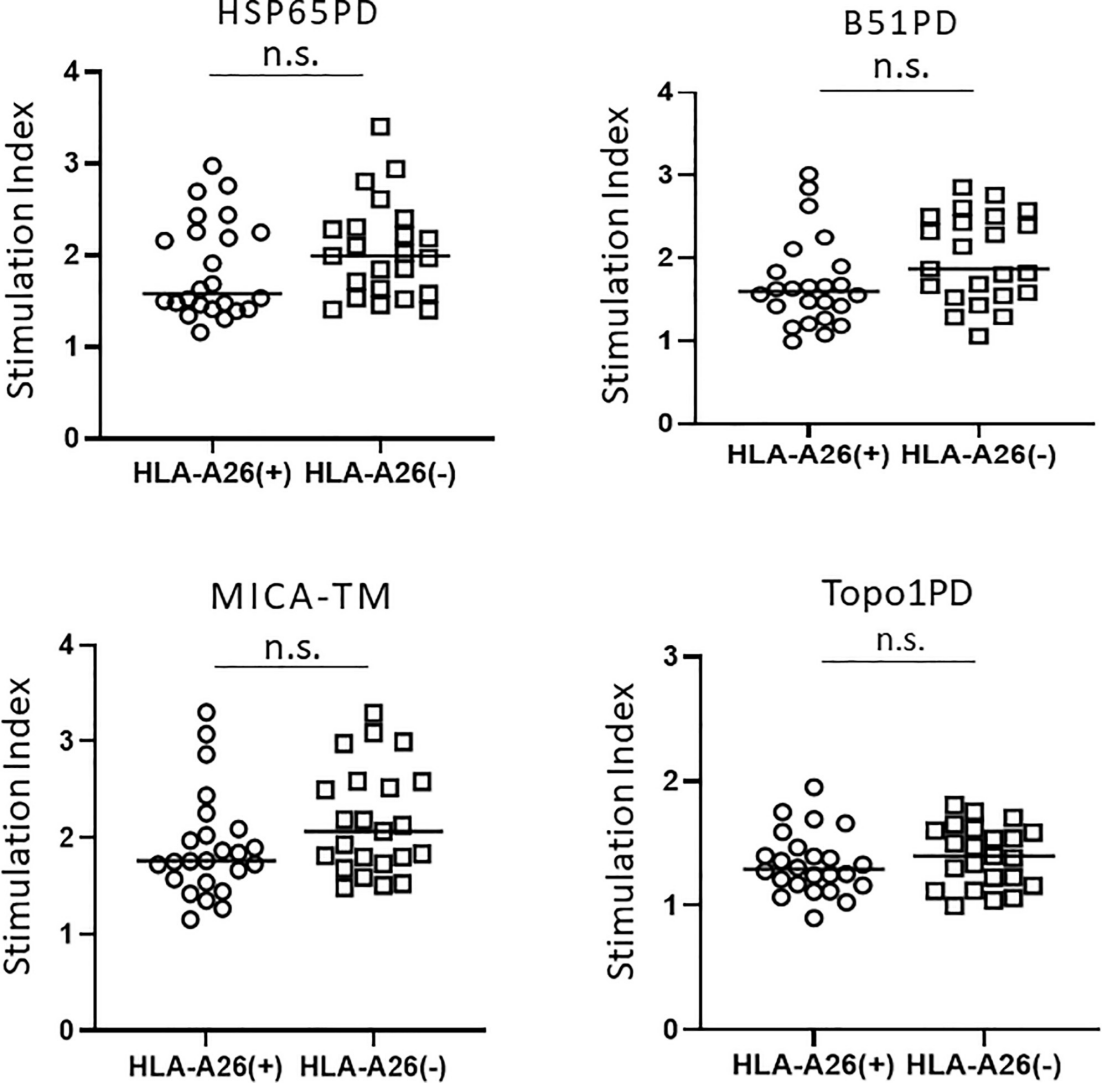
Association of HLA-A*26 and T cell proliferation in Behçet’s disease. Lymphocyte proliferative responses in Behçet's disease induced by four peptides (HSP65, B51PD, MICA-TM, and Topo1PD) were compared between HLA-A*26-positive patients and HLA-A*26-negative patients. The SI for each group is shown. The statistical significances between BD and other diseases were determined using Mann–Whitney’s U-test. n.s., Not significant; HLA, human leukocyte antigen.

We assessed the association of HLA-A*26 and T cell proliferation induced by candidate peptides (Fig 3). There were no significant differences in PBL proliferation between HLA-A*26-positive and -negative BD groups when the PBL were stimulated by all four peptides (n.s., Mann–Whitney’s U-test).

## Discussion

Although the etiology of BD is not clear, the association of autoreactive T lymphocytes and the increase of Th1/Th17 cells and decrease of regulatory Treg cells was speculated as one of the causes of recurrent uveitis [6]. Recently, several antigens and their peptides have been assessed as candidate autoantigens associated with BD with uveitis [4,7–12].

Recently, using molecular dynamics simulation, we demonstrated that MICA-TM, one of the candidate peptides that induced proliferation of PBL from patients with BD, had a significantly stronger total binding free energy with the BD-associated HLA alleles (HLA-B*51:01 and A*26:01) than with the non-associated alleles (HLA-B*35:01 and A*11:01) [21]. Because molecular dynamics simulation can suggest actual molecular binding energies between peptides and HLA receptors, we speculate that this method will be a useful tool for selecting candidate peptides that can induce higher proliferative response in PBL from patients with T cell-mediated inflammatory diseases associated with specific HLA alleles. However, further analysis is required to confirm this prediction.

In the present study, we examined the proliferative response of PBL from patients with BD and other diseases to HSP65PD and B51PD. These peptide sequences had high-affinity to the HLA-B*51:01 binding pocket as observed using computerized binding predictions (IEDB analysis resource Consensus tool) [15]. The results of this study clearly demonstrated that these two peptides can induce significantly higher proliferation of PBL from patients with BD than from HC, whereas proliferation of PBL from patients with other diseases (sarcoidosis, VKH, and SSc) was similar to that of HC (Fig 1). Notably, the proliferative reaction of PBL was HLA-B*51:01 dependent; it was significantly stronger in HLA-B*51:01-positive BD patients than in HLA-B*51:01-negative BD patients (Fig 2). In contrast, there were no significant differences in lymphocyte proliferation between HLA-A*26-positive BD patients and HLA-A*26-negative BD patients (Fig 3). This suggested that the higher affinity of the peptide to the HLA binding pocket might cause stronger lymphocyte proliferation. As this study investigated only one HLA epitope (HLA-B*51:01), further studies on other HLA epitopes and diseases are necessary to confirm this hypothesis. Furthermore, sequencing the peptides eluted from HLA-B51 positive antigen-presenting cells in patients with BD and control groups may help confirm the current results, although this may be challenging.

The control peptide Topo1PD was a 9-mer peptide from topoisomerase 1, an antigen highly associated with SSc, as anti-topoisomerase 1 antibody (anti-Scl-70) was detected in the sera of 20−70% of patients with SSc [31]. In a previous report, a 15-mer peptide for topoisomerase 1 induced significantly strong proliferation of PBL from patients with anti-topoisomerase 1 antibody-positive SSc [29]. However, the Topo1PD peptide, a partial 9-mer peptide of the 15-mer peptide used in the present study, did not induce a significant increase in PBL proliferation in either BD or SSc. Although the underlying reason for this observation was unclear, one of the main reasons may be that only 26.7% (4/15) cases of SSc were anti-topoisomerase 1-positive in the present study. Further studies with higher numbers of anti-topoisomerase 1-positive SSc patients are required to clarify this issue.

The reason why both HLA- B51 negative and B51 positive subjects responded in this study is unclear and may be difficult to explain. Potential reasons include: (1) there exist HLA class I types other than HLA-B51 with high binding ability to these peptides, (2) HLA class 2 (HLA-DR) might play a role in lymphocyte proliferation by these peptides, or (3) receptors other than HLA, such as Toll-like receptors, might be involved in lymphocyte proliferation.

The major strength of this study was that all experiments were performed at the University of Tokyo, which allowed maintenance of a stable experimental system and yielded reproducible results. However, this study has several limitations. First, the number of patients with sarcoidosis, VKH, SSc, and HC was low. Second, the age and sex distribution of HC and patients with sarcoidosis, VKH, and SSc were slightly different; the age and sex distribution of HC matched with those of patients with BD in this study, whereas the patients with BD and HC were relatively younger than those with sarcoidosis, VKH, and SSc. Furthermore, the female to male ratio was high among patients with SSc in Japan. Third, no inclusion criteria were set regarding current medical therapy or severity of disease, which may affect the individual SI values. Fourth, lymphocyte proliferation in response to an antigen does not prove the contribution of the antigen to pathogenesis. Despite these limitations, we believe that the present findings provide new and important insights regarding the benefits of the recent computerized binding predictions [15] for the selection of peptide epitopes specific for HLA molecule, which might induce higher proliferation of PBL.

## Conclusions

In conclusion, two peptides with high affinity to HLA-B*51:01 were identified using computerized binding predictions [15]. These peptides showed significantly high response in HLA-B*51:01-positive patients with BD. The recent computerized simulations for predicting affinity between peptides and HLA pockets might be useful for determining autoreactive peptides for HLAs.

## Supporting information

S1 Fig Difference in lymphocyte proliferation by sex in patients with Behçet’s uveitis. Lymphocyte proliferation induced by candidate peptides was compared by sex using Mann–Whitney’s U-test. n.s., not significant.
(TIF)

S1 Table Dataset. Minimal dataset of lymphocyte proliferation in patients with Behçet's uveitis.
(XLSX)

S2 Table Dataset. Minimal dataset of lymphocyte proliferation in patients with control diseases and healthy controls.
(XLSX)
