# Supplementary figures and images for: Lymphocyte proliferation induced by high-affinity peptides for HLA-B*51:01 in Behçet’s uveitis
Source: PLoS One. 2019 Sep 12;14(9):e0222384. doi: 10.1371/journal.pone.0222384 (PMC6742369; doi:10.1371/journal.pone.0222384)

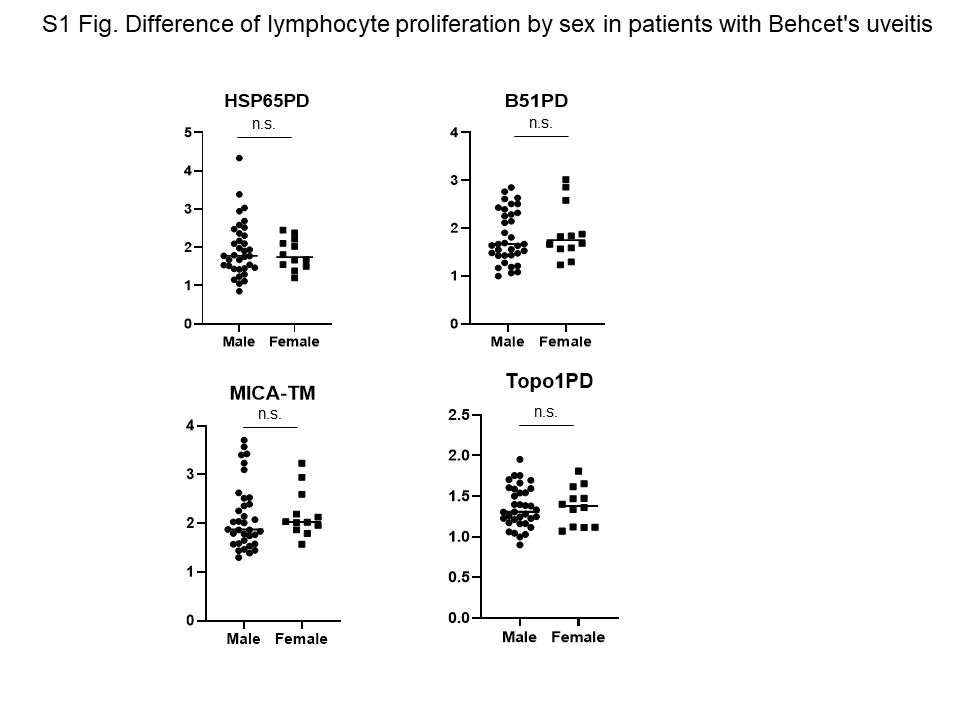

Supplement: S1 Fig — Lymphocyte proliferation induced by candidate peptides was compared by sex using Mann–Whitney’s U-test. n.s., not significant. (TIF) [file pone.0222384.s001.tif]
